# Transmission of Urgency Levels in the Alarm Calls of the Oriental Reed Warbler

**DOI:** 10.1002/ece3.72338

**Published:** 2025-11-12

**Authors:** Qindong Zhou, Laikun Ma, Wei Guo, Jiaojiao Wang, Longwu Wang

**Affiliations:** ^1^ School of Life Sciences Guizhou Normal University Guiyang China; ^2^ Department of Biology and Food Science Hebei Minzu Normal University Chengde China; ^3^ College of Life Science Hebei University Baoding China; ^4^ Engineering Research Center of Ecological Safety and Conservation in Beijing‐Tianjin‐Hebei (Xiong'an New Area) of MOE Baoding China; ^5^ Hebei Basic Science Center for Biotic Interaction Baoding China; ^6^ Ministry of Education Key Laboratory for Ecology of Tropical Islands, Key Laboratory of Tropical Animal and Plant Ecology of Hainan Province, College of Life Sciences Hainan Normal University Haikou China

**Keywords:** alarm calls, conspecific communication, oriental reed warbler, playback experiment

## Abstract

Many animals communicate predator‐related information to conspecifics through alarm calls that exhibit acoustic structural variations encoding key parameters such as predator type, body size, and urgency of danger. In previous studies, the oriental reed warbler ( *Acrocephalus orientalis* ) produced a variety of syllable types in its alarm calls when encountering three species of invaders—Eurasian sparrowhawk ( *Accipiter nisus* ), common cuckoo ( *Cuculus canorus* ), and oriental turtle dove ( *Streptopelia orientalis* ). However, the information conveyed by its alarm calls does not include the type or size of the predator. To explore whether these different syllable types convey varying levels of urgency, we conducted playback experiments during the oriental reed warbler's incubation period. Three syllable types with gradient differences in the acoustic parameters were selected: B alarm calls, D alarm calls, and F alarm calls. The results indicate that the oriental reed warbler responds differently to different types of alarm calls. Specifically, the oriental reed warbler exhibited a stronger response to F alarm calls than to B alarm calls, manifested by higher response intensity, increased attack frequency, and greater individual attraction. In addition, the urgency scores triggered by F alarm calls were significantly higher than those triggered by B and D alarm calls. Furthermore, the frequency and duration of different syllable types significantly predicted conspecific responses. Our results suggest that oriental reed warblers utilize higher‐frequency calls with longer durations in their alarm signals to convey a greater sense of urgency regarding danger. Further synthetic sound experiments are needed to investigate the relative significance of duration and frequency, and to determine the role of syllable sequence in conveying predator‐specific information.

## Graphical abstract

Previous studies revealed that oriental reed warblers ( *Acrocephalus orientalis* ) produce alarm calls containing diverse syllable types in response to intruders. We selected three distinct syllable types from these calls (B, D and F alarm calls) for playback experiments to test whether different syllable types convey graded urgency information. Our study found that the Oriental Reed Warbler exhibited the highest level of responsiveness to F alarm calls, characterised by the most pronounced response intensity and the greatest aggregation of individuals. In contrast, no significant differences were identified between alarm calls B and D. These findings suggest that the Oriental Reed Warbler may not be able to use a linear hierarchical framework to encode threat information.

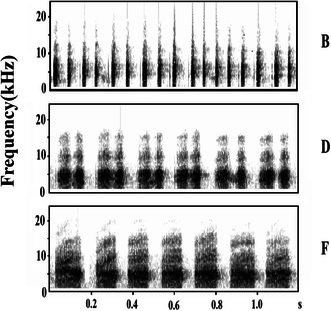

## 1 Introduction

Breeding is a critical phase in the life cycle of avian species, with reproductive success contingent upon a variety of disperturbing factors, particularly predation and parasitism (Woodworth 1997; Ibáñez‐Álamo et al. 2015). Avian species have developed a range of defence strategies to safeguard themselves and their nests against intruders. For instance, host organisms can enhance reproductive success through techniques such as the recognition of eggs (Wang et al. 2015) and chicks (Langmore et al. 2003; Noh et al. 2018). When encountering brood parasitism, host birds initiate nest defence behaviours and produce alarms (Yu et al. 2016, 2019). When confronted by predators, birds adopt behaviours such as camouflage (Koskenpato et al. 2020), sounding alarms (Dutour et al. 2019), vigilance (Jiang et al. 2021), distraction (Smith 2017), or escape (Morelli et al. 2019).

Alarm calls serve as a crucial medium for avian risk communication, playing a pivotal role in the defensive behaviours of nearly all bird species. Alarm calls are typically defined as open social signals, with one of their primary functions being to convey information about threats to relatives or other group members (Otter 2007; Magrath et al. 2015). The vocal system of birds exhibits intricate structural characteristics that enable them to convey a substantial volume of information, allowing the receiver to respond to potential threats (Evans 1997). Variations in the acoustic characteristics of alarm calls enable birds to communicate information regarding the type, size, velocity, and behaviour of predators (Zuberbühler 2009; Gill and Bierema 2013; Manser 2009). For instance, yellow warblers ( *Setophaga petechia* ) produce a “seet” vocalisation in response to parasitic threats and a “chip” vocalisation to predator threats (Gill and Sealy 1996). The Carolina chickadee ( *Poecile carolinensis* ) employs alarm calls with distinct syllable types in response to the presence of high‐risk terrestrial or aerial predators (Freeberg 2008; Soard and Ritchison 2009). Conversely, singular alarm calls can vary in the syllable count per call, call frequency, or call duration. For instance, the calls of white‐browed scrubwren ( *Sericornis* , Leavesley and Magrath 2005) increase in syllable count as a predator approaches, while the American crow ( *Corvus brachyrhynchos* ) produces longer duration, higher frequency, and shorter interval alarm calls in response to high‐risk predators (Yorzinski and Vehrencamp 2009). Furthermore, avian species can communicate information regarding threats through various modalities, including discrete signals, graded responses, and the arrangement and combination of distinct calls and syllable types in (Yu et al. 2016; Templeton et al. 2005; Wilson and Evans 2012). For example, great tits ( *Parus major* ) produce alarm calls with distinct acoustic properties depending on the predator. Specifically, they produce a ‘jar’ call in response to snakes and a ‘chicka’ call when faced with crows or martens. Furthermore, they differentiate crows and martens by altering the calling rate and number of the ‘chicka’ calls (Suzuki 2014).

The oriental reed warbler ( *Acrocephalus orientalis* ) is the preferred host of the common cuckoo ( *Cuculus canorus* ) in China and has undergone coevolution with this parasitic species (Yang et al. 2014; Li et al. 2016). In the nest defence phase, the oriental reed warbler exhibits varied responses to different types of nest invaders, indicating that it can distinguish between cuckoos and other intruders based on visual cues (Li et al. 2015; Ma, Yang, and Liang 2018; Wang et al. 2021). For instance, Wang et al. (2021) documented that oriental reed warblers exhibited intense aggressive responses toward common cuckoo specimens, demonstrating significantly greater attack intensity against cuckoo models compared to sparrowhawk ( *Accipiter nisus* ) and oriental turtle dove ( *Streptopelia orientalis* ) specimens. Complementing this, Li et al. (2015) quantified that adult oriental reed warblers' attack intensity decreased significantly across stimulus types: from common cuckoo stuffed dummies (8.50 ± 1.84) to dove (3.20 ± 0.70), sparrowhawk (2.34 ± 0.52), and magpie ( *Pica pica* ) stuffed dummies (0.56 ± 0.13). However, the alarm calls of the oriental reed warbler in response to nest intruders do not seem to convey information regarding the nature of the threat. Indeed, a previous study revealed that the oriental reed warbler does not vary its alarm calls significantly by intruder type, indicating an absence of specific referential signalling (Wang and Yang 2020; Wang et al. 2021). However, the oriental reed warbler has been observed to produce six distinct types of alarm call syllables. Whether these syllables elicit differing responses in oriental reed warblers requires further investigation.

This study examined three distinct syllable types of alarm calls from the oriental reed warbler: B alarm calls, D alarm calls, and F alarm calls. By conducting playback experiments during the incubation period, we aimed to determine whether these syllable types elicit correspond to a graded defensive response. Yorzinski and Vehrencamp (2009) revealed that the American crows, which mainly inhabit the North American continent, convey more severe danger information by emitting mobbing calls that are of longer duration, higher frequency, and shorter intervals. Guided by prior studies, we predicted the oriental reed warbler would exhibit the lowest defensive response to the B alarm call, a moderate defensive response to the D alarm call, and the greatest defensive response to the F alarm call.

## 2 Methods

### 2.1 Study Site and Subjects

The study was carried out from May to July in both 2024 and 2025 at Yongnianwa National Wetland Park, located in Handan City, Hebei Province, China (coordinates: 36°40′–36°41′ N, 114°41′–114°45′ E). Yongnianwa National Wetland Park is an inland freshwater wetland ecosystem characterised by significant biodiversity. The park encompasses a total area of 1070.4 ha and is situated within a temperate semi‐humid continental monsoon climate zone at 41 m above sea level. The vegetation in lowland areas is diverse, with extensive populations of reeds, irises, and lotus flowers (Ma, Yang, Liu, et al. 2018). The oriental reed warbler is a small passerine species within the order Passeriforms (Zheng 2023). This species typically breeds in reed habitats and has demonstrated a robust population in the wetland park. An average of 265.33 ± 31.16 (Mean ± SD) nests has been recorded over the past three years (personal observation). The oriental reed warbler is one of the principal hosts of the common cuckoo, with a parasitism rate of approximately 14.8% in this area (Ma, Yang, Liu, et al. 2018). The local predatory fauna comprises snakes, Siberian weasels ( *Mustela sibirica* ), oriental magpies (*Pica serica*), and various raptor species.

### 2.2 Production of Playback Sound

**FIGURE 1:**
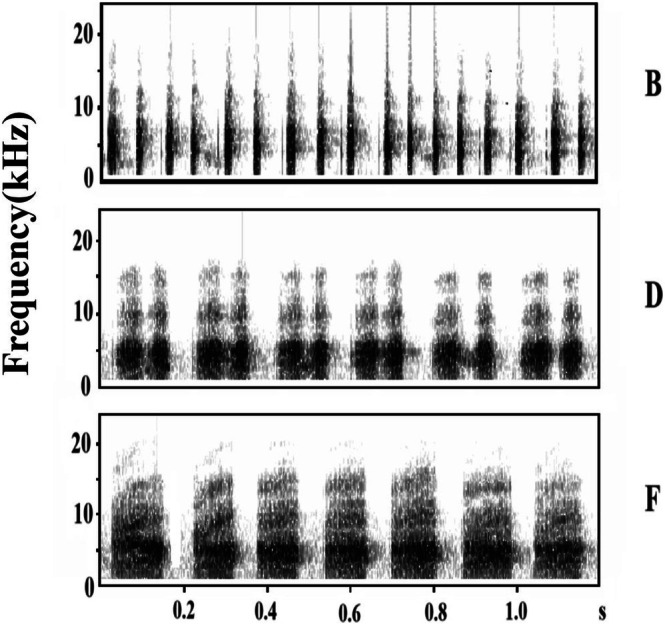
Spectrograms of three types of alarm calls (B, D, and F).

A previous experiment at this study site that sequentially recorded alarm calls produced by the oriental reed warbler in response to specimens of the common cuckoo (parasite), Eurasian sparrowhawk (a predator), and oriental turtle dove (control) indicated no significant differences in the alarm calls (Wang and Yang 2020). However, the alarm calls of the oriental reed warbler include six types of syllables (Wang et al. 2021). In this study, three distinct syllable types were selected from oriental reed warbler alarm calls directed at different intruder types: B, D, and F alarm calls (Figure 1). These vocalisations originated from four individual warblers and were then imported into Raven Pro 1.4 (Cornell Laboratory of Ornithology, Ithaca, NY, USA). All stimuli were constructed de novo by concatenating conspecific alarm calls of identical syllable types into 1‐min WAV files. For each of the three call types (B alarm call, D alarm call, and F alarm call), two distinct stimulus sets were generated. During compilation, overlapping vocalisations and ambient noise below 0.2 kHz were rigorously excluded while preserving the original temporal structure of vocalisations (Wilson and Mennill 2011; Suzuki 2012). As shown in Figure 1, spectrograms characterise the acoustic structure of all three alarm call variants (B, D, and F alarm calls). Additionally, a background noise file was created using the same methodology as the alarm call files, which served as a control. The playback volume was calibrated to approximately 75 dB 1 m from the speaker (Yu, Lv, et al. 2017; Yu et al. 2019).

### 2.3 Playback Experiment

The playback experiment was conducted in the Yongnianwa National Wetland Park of Hebei Province from May to July 2024 and June 2025. A total of 34 nests were subjected to 75 playback sessions. All trials occurred under clear, windless conditions (wind speed < 1 m/s) within specified time windows (06:30–11:30 or 14:00–18:00). Playback experiments were conducted during the incubation period of oriental reed warblers. To avoid or reduce pseudoreplication, a total of 6 alarm calls from 4 nests were used (two each of B, D, and F alarm calls). The interval between each trial exceeded one hour, and all trials within the same nest were completed within a single day. For some nests where the trials could not be completed in one day due to factors such as weather conditions or human disturbance, the remaining trials were conducted on the following day (Yu, Xing, et al. 2017).

During the experiments, a Bluetooth player (P2, Shidu Digital Technologies Inc., Shenzhen, China) was fixed on reeds within a 1m radius of the nest. The camera (WJO3, HiSilicon Technologies Inc., Shanghai, China) was set up 5 m from the nest to center the nest in the frame and cover a ≥ 2‐m observational radius. We connected to the playback device via mobile Bluetooth, and the observer remained quiet approximately 5 m from the nest. Playback experiments began when no surrounding birds were issuing alarm calls. Each experiment was played back at the same volume level. All behavioural responses were recorded by one person (Q.Z.) to avoid differences between observers.

Each experimental session lasted 6 min in total, consisting of a 5‐min settling period (to eliminate the influence of human disturbance on subject behaviour) and a 1‐min playback period. We revisited the nest 2 days after the experiment and did not observe any signs of nest abandonment by the adult birds. Behavioural responses were recorded for 1‐min upon playback initiation, with recorded behavioural responses including: (1) response intensity (maximum response intensity within 1 min): scored as watching (bird goes straight back to brood or ignores the sound without further action; score = 1), alert (bird exhibits rapid flight patterns around the playback device, testing it with its beak or concealing itself while emitting alarm calls without displaying aggressive behaviour; score = 2), mobbing (bird emits alarm calls and performs a feigned attack on the playback device; score = 3), and attack (bird emits alarm calls and physically contacts the playback device; score = 4); (2) response latency(s): time from the onset of the playback to the moment the focal bird left the nest; (3) number of individuals attracted (n): count of conspecific individuals attracted; (4) number of attacks (n): number of times adult birds struck the speaker with their bodies during the 1 min sound playback period (Yu, Lv, et al. 2017; Wang et al. 2021); (5) urgency score: the overall urgency level of focal individuals, determined by first normalising four behavioural response variables—latency to respond, response intensity, number of individuals attracted, and number of attacks—using the Min‐Max normalisation method. As a shorter latency implies higher urgency, we applied reciprocal transformation during standardisation. Subsequently, we calculated the weight of each indicator using the entropy weighting method and derived a composite urgency score for each trial.

### 2.4 Sound Analysis

We imported the previously recorded alarm calls (B alarm call, *n* = 10; D alarm call, *n* = 10; F alarm call, *n* = 10) into Raven Pro 1.4 (Cornell Laboratory of Ornithology, Ithaca, NY, USA) and only analysed the low‐noise non‐overlapping alarm calls (Courter and Ritcisson 2010; Suzuki 2014). Referencing the literature (Davies et al. 2006; Samaš et al. 2020), we selected several parameters commonly used in sound measurement (Table 1), including (1) low frequency; (2) high frequency; (3) peak frequency; (4) delta time; (5) delta frequency; (6) inter‐onset interval; (7) call rate (Suzuki 2014).

**TABLE 1:** Descriptive statistics of the sound parameters of different alarm calls used in playback experiments (Mean ± SD).

Type	Low frequency (Hz)	High frequency (Hz)	Peak frequency (Hz)	Delta time (Hz)	Delta frequency (Hz)	Inter‐onset interval (s)	Call rate (s)
B	792.32 ± 228.33	17977.69 ± 2256.99	3924.77 ± 371.86	0.02 ± 0.00	17185.37 ± 2247.06	0.1 ± 0.24	22.84 ± 3.95
D	704.34 ± 312.96	19632.91 ± 3284.56	4083.13 ± 686.78	0.05 ± 0.01	18928.57 ± 3577.84	0.05 ± 0.02	11.89 ± 2.11
F	558.38 ± 215.09	21416.29 ± 1259.46	4470.67 ± 392.6	0.14 ± 0.02	20857.91 ± 1394.17	0.11 ± 0.09	4.7 ± 0.81

### 2.5 Statistical Analyses

Statistical analyses were performed using R 4.3.1 and IBM SPSS Statistics 27.0. We extracted seven acoustic parameters from the original playback recording using Raven Pro 1.4 (Table 1), and principal component analysis (PCA) was used to extract the major principal components (PCs) from the original sound parameters. Then, we used the K‐W test in non‐parametric tests to analyse the differences in sound parameters among different playback types. Linear regression was used in the generalised linear mixed model (GLMM) to fit the relationship between the extracted principal components and the urgency score. The background noise control group followed an identical experimental protocol to that of the alarm call treatments (6 min per nest, including a 5‐min cooling‐off period and 1‐min playback period). Video analysis confirmed that adult oriental reed warblers exhibited no defensive behaviours in response to background noise playback. As no behavioural changes were observed in the background group, this group was excluded from the modelling analysis.

For the ordinal response variable “response intensity”, we constructed a cumulative link mixed model (CLMM) using the “ordinal” package in R 4.3.1. We used the logit‐link function to predict the reaction intensity (1 = watching, 2 = alert, 3 = mobbing, 4 = attack) of the oriental reed warbler to different playback stimuli (B alarm call, D alarm call, and F alarm call) and used the two‐tailed likelihood ratio test to obtain the *p*‐value. When significant differences between the types of playbacks were observed, we conducted additional CLMM. Generalised linear mixed models (GLMMs) with a negative binomial error structure and log link function were used to analyse the other behavioural responses, including response latency, number of individuals attracted, number of attacks, and urgency score. In all models, nest ID was included as a random effect, playback type was entered as a fixed factor, and clutch size and days since hatching were included as covariates. In GLMMs, *p*‐values for pairwise comparisons were adjusted using the sequential Bonferroni method. All tests were two‐tailed, and the significance threshold was set at *p* = 0.05.

## 3 Results

A total of 75 playback trials were conducted across 34 nests, including 25 trials each for the B, D, and F alarm calls. The seven sound parameters extracted for different playback types showed significant differences in general (all *p* < 0.05, K–W test). PCA of the original acoustic parameters yielded two components (PC1 and PC2; eigenvalues > 1.0), which together explained 73.044% of the total variance. Factor loadings for the principal components are listed in Table 2.

**TABLE 2:** Principal component analysis of acoustic parameters identifying key components associated with the urgency score in the playback experiments.

	PC1	PC2
Acoustic parameter		
Low frequency	−0.813	−0.119
High frequency	0.883	0.298
Peak frequency	0.676	0.277
Delta time	0.288	0.89
Delta frequency	0.902	0.291
Inter‐onset interval	−0.531	0.361
Call rate	−0.161	−0.908

The principal components PC1 (*H* = 11.891, df = 2, *p* = 0.003) and PC2 (*H* = 57.018, df = 2, *p* < 0.001) of different playback types have shown significant differences. Overall, playback type had a significant effect on response intensity (*χ* ^*2*^ = 14.261, df = 2, *p* = 0.0008), the number of individuals attracted ($F_{2,70}$ = 6.190, *p* = 0.003; Table 3), and the number of attacks ($F_{2,70}$ = 4.477, *p* = 0.015; Table 3). Playback type also significantly influenced the overall urgency score ($F_{2,70}$ = 3.655, *p* < 0.0001; Table 3). However, the playback types did not have a statistically significant effect on the response latency ($F_{2,44}$ = 3.884, *p* = 0.212; Table 3). Hatching day had no significant effect on any of the behavioural responses (all *p* > 0.05; Table 3). Clutch size significantly affected the number of individuals attracted ($F_{1,70}$ = 4.283, *p* = 0.042; Table 3), while its effects on other response variables were not significant (all *p* > 0.05; Table 3).

**TABLE 3:** Statistical analysis results of the oriental reed warbler defence level (response variable) under different playback stimuli.

Effects	*F*	df1	df2	*p*		*F*	df1	df2	*p*
Response latency (*n* = 25)					Number of individuals attracted (*n* = 25)				
Hatching day	0.668	1	44	0.418	Hatching day	1.161	1	70	0.285
Clutch size	0.369	1	44	0.547	Clutch size	4.283	1	70	0.042*
Type of playback	3.884	2	44	0.212	Type of playback	6.190	2	70	0.003**
Number of attacks (*n* = 25)					Urgency score (*n* = 25)				
Hatching day	0.382	1	70	0.538	Hatching day	0.706	1	70	0.391
Clutch size	0.448	1	70	0.505	Clutch size	1.133	1	70	0.291
Type of playback	4.477	2	70	0.015*	Type of playback	8.615	2	70	< 0.001***

* *p* < 0.05.

** *p* < 0.01.

*** *p* < 0.001.

**FIGURE 2:**
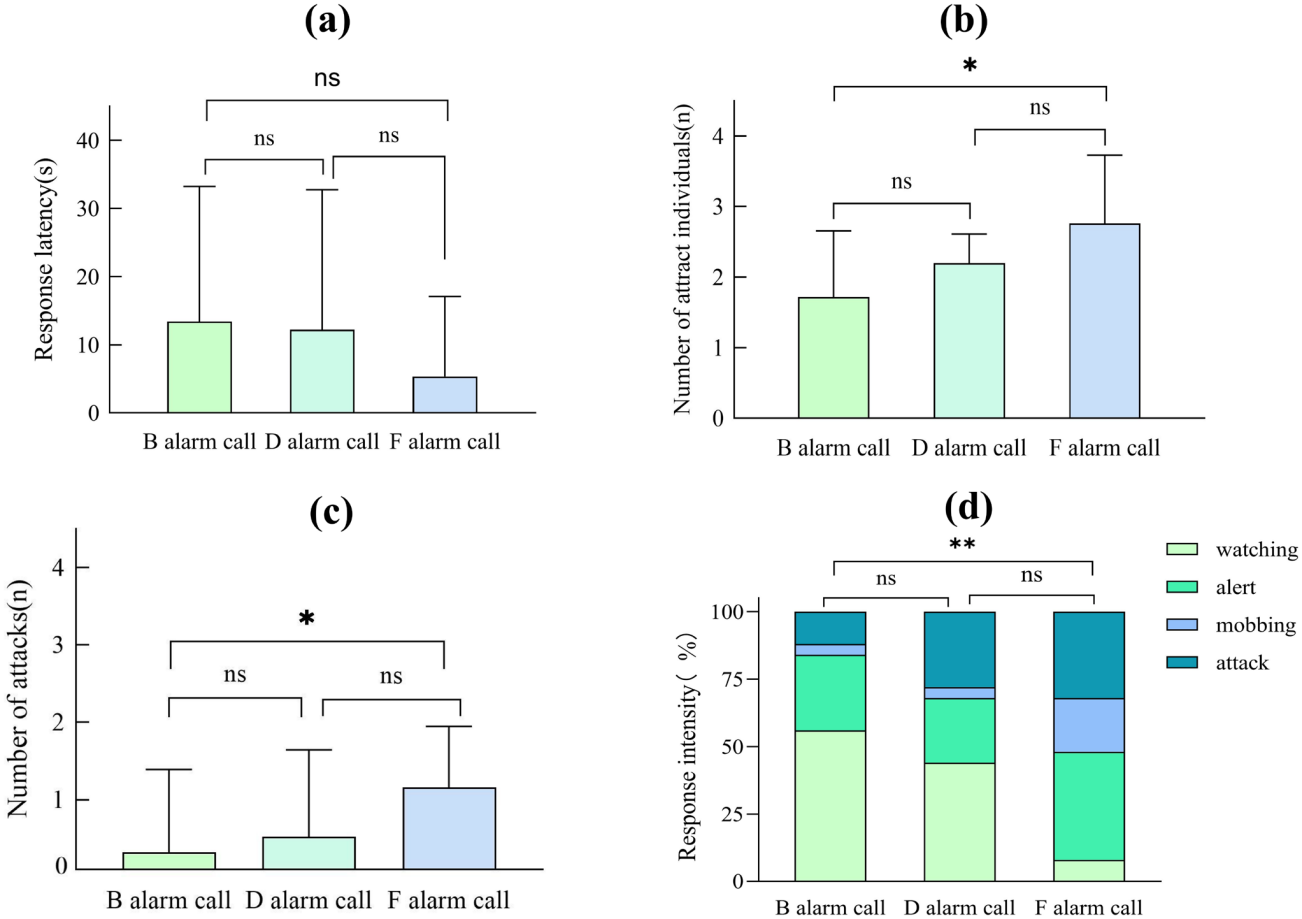
Pairwise comparisons of behavioural responses of oriental reed warblers under different playback treatments. Each panel shows the mean (±SD) of a specific behavioural variable: (a) response latency, (b) number of individuals attracted, (c) number of attacks, and (d) response intensity. Asterisks denote statistically significant differences. ^ns^ *p* > 0.05, *p < 0.05, **p < 0.01.

**FIGURE 3:**
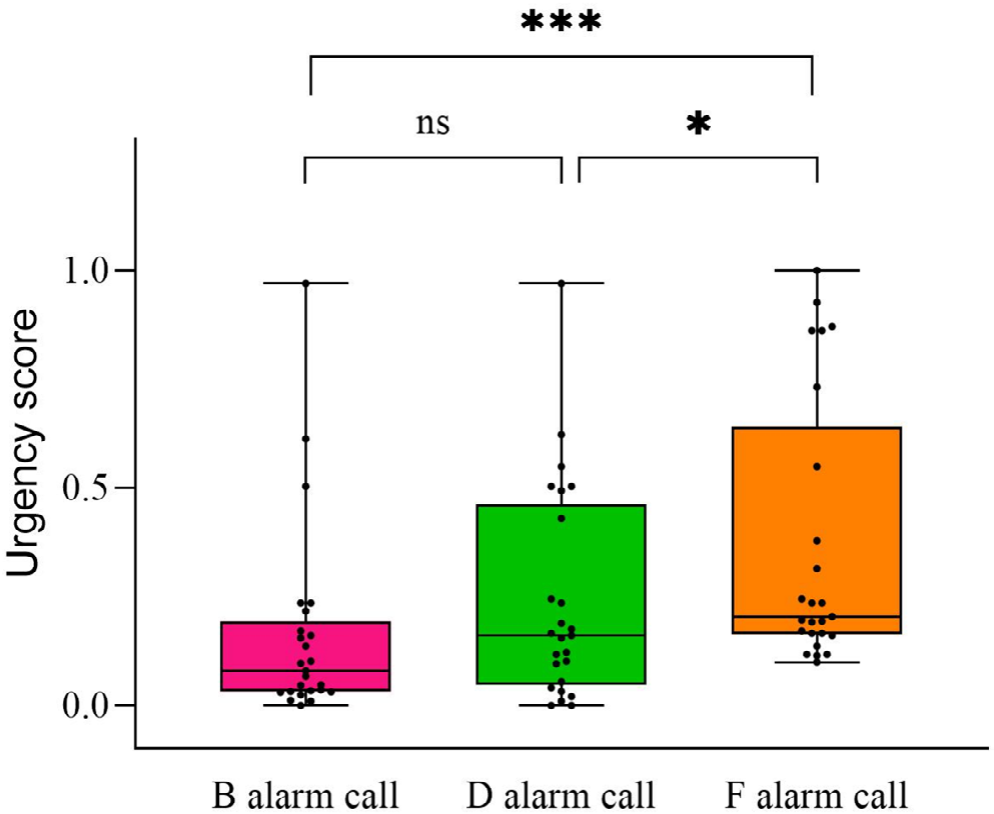
Differences in the overall urgency level (presented as scores) of oriental reed warblers among different playback types. *p < 0.05; ***p < 0.001.

Post hoc comparisons revealed that the response intensity elicited by F alarm calls was significantly higher than that of B alarm calls (*z* = 3.321, *p* = 0.003; Figure 2, Table 4). However, no significant differences were found between F and D alarm calls (*z* = 2.179, *p* = 0.075; Figure 2, Table 4) or between B and D alarm calls (*z* = 1.316, *p* = 0.386; Figure 2, Table 4). F alarm calls also attracted significantly more individuals than B alarm calls (*t* = 2.652, *p* = 0.030; Figure 2) and prompted significantly more attacks (*t* = 2.809, *p* = 0.019; Figure 2). No other statistically significant pairwise comparisons were observed (*p* > 0.05; Figure 2). Regarding the urgency score, F alarm calls elicited the highest urgency level, significantly greater than that of B alarm calls (*t* = 4.055, *p* < 0.001; Figure 3) and D alarm calls (*t* = 2.663, *p* = 0.019; Figure 3). There was no significant difference in the urgency score between B alarm calls and D alarm calls (*t* = 1.229, *p* = 0.223; Figure 3). We also found that both PC1 and PC2 of the extracted sound parameters significantly affected the urgency score (PC1: *t* = 2.452, *p* = 0.017; PC2: *t* = 0.887, *p* = 0.005; Table 5).

**TABLE 4:** Statistical analysis results of oriental reed warbler response intensity under different playback stimuli.

Effect	Estimate	SE	*z*	*p*
Hatching day	−0.035	0.134	−0.263	0.793
Clutch size	0.121	0.371	0.326	0.745
B vs. D	0.849	0.645	1.316	0.386
B vs. F	2.256	0.679	3.321	0.003**
D vs. F	1.407	0.645	2.179	0.075

** *p* < 0.01.

**TABLE 5:** Linear regression results for urgency scores across different playback treatments.

Effect	Coefficient	SE	*t*	*p*	95% CI
Intercept	0.202	0.305	0.663	0.51	[−0.406 ~ 0.811]
Hatching days	−0.013	0.142	−0.929	0.356	[−0.111 ~ 0.591]
Clutch size	0.036	0.455	0.781	0.437	[−0.055 ~ 0.126]
Frequency PC1	0.049	0.202	2.452	0.017*	[0.009 ~ 0.090]
Temporal PC2	0.067	0.232	0.887	0.005**	[0.021 ~ 0.113]

* *p* < 0.05.

** *p* < 0.01.

## 4 Discussion

Previous studies have demonstrated that the acoustic structure of alarm calls—such as call type, call rate, syllable combinations, and element number—enables animals to convey information about the level and nature of predation risk (Manser et al. 2002; Zuberbühler 2009; Wilson and Mennill 2011; Gill and Bierema 2013; Suzuki 2014; Yu, Lv, et al. 2017). However, our previous research on the oriental reed warbler revealed no significant variation in alarm call types emitted toward different nest intruders. Notably, six distinct syllable types have been identified within the species' alarm repertoire (Wang et al. 2021), raising the question of whether these syllables encode graded or categorical threat‐related information.

Alarm calls in animals generally reflect the presence of predators or other urgent threats and convey urgency through variation in acoustic structure, such as duration, syllable shape, and repetition rate (Suzuki 2016). For instance, black‐capped chickadees ( *Poecile atricapillus* ) alter the repetition rate of the “D” (or “dee”) notes in their “chick‐a‐dee” calls to encode different types of threat information (Templeton et al. 2005). Similarly, in the white‐throated magpie‐jay ( *Calocitta formosa* ), both syllable rate and duration are correlated with the level of perceived (Ellis 2008). Our findings demonstrate a strong correlation between the behavioural responses of oriental reed warblers and specific acoustic features, particularly delta frequency, syllable rate, and syllable duration. The warblers' heightened responses were more likely driven by variation in acoustic parameters—such as increased frequency or extended syllable duration—rather than by the identification of predator type. This pattern is consistent with Griesser (2009), who showed that Siberian jays ( *Perisoreus infaustus* ) increased call rates under high‐risk conditions to convey stronger threat signals to group members. Our data indicate that the alarm calls of the oriental reed warbler are characterised by longer durations and higher frequencies, which may serve as key acoustic markers of escalating threat perception.

In this study, playback experiments revealed that alarm calls effectively attracted conspecifics near the focal nest in most cases (70 out of 75 trials). Notably, neighbouring individuals responded rapidly to F alarm calls, with eight individuals (8/25) physically attacking the speaker (the focal bird's attack on the playback device is not a natural behaviour in the studied species; it is a response to the alarm call itself rather than a reaction toward the individual producing the call). Furthermore, 21 out of 25 birds approached the playback site within 5 s, a pattern resembling the function of “mobbing calls”, in which conspecifics are recruited to collectively repel intruders, which has the function of warning and threatening, deterring predators (Dutour et al. 2024). F alarm calls elicited shorter mean response latency (5.36 ± 11.935 s) than B and D alarm calls. Our analysis of acoustic parameters and urgency scores in oriental reed warblers revealed that alarm calls containing higher frequencies and longer durations elicited stronger conspecific responses. This finding suggests that F alarm calls may transmit more urgent and socially recognisable threat information, prompting neighbouring individuals to adopt contextually appropriate defence strategies. Such rapid approach behaviour may facilitate cooperative defence at the nest site, ultimately enhancing reproductive success and survival. Therefore, the urgency coding of alarm calls in the oriental reed warbler may represent an adaptive element of its defensive strategy.

Contrary to our initial hypothesis, the behavioural responses to B and D alarm calls did not differ significantly in intensity. This result suggests that threat‐related information in this species may not follow a strictly linear gradation model, wherein increasing acoustic features correspond directly with increasing behavioural responses. Instead, a threshold‐based system may be at play, in which only signals that surpass a certain acoustic threshold elicit marked responses. Due to experimental limitations—specifically, the absence of all six syllable types in simultaneous playback—we were unable to fully test the presence of a nonlinear threshold encoding system.

Further research is required to clarify several important questions. For example, playback experiments using artificially synthesised alarm calls—featuring combinations of shorter syllable duration with higher frequency, or longer duration with higher frequency—could help identify which acoustic features play a key role in threat communication. It would also be informative to investigate whether alarm calls with short duration and low frequency function as initial, low‐urgency signals intended to alert mates rather than to recruit group members. Additionally, the sequential order of syllables within alarm call bouts may carry additional contextual or referential information, and the proportion of F‐type syllables within a call sequence is also a potentially important factor. Therefore, future studies should focus on syllable structure, combinatorial order, and relative proportions within natural alarm call sequences to elucidate how acoustic features shape defensive behaviour. Finally, it is also unclear whether individuals respond differently to alarm calls from their mate compared to those from unfamiliar individuals, requiring further investigation. Such work will advance our understanding of the vocal encoding of threat in birds and contribute to broader insights into the evolution of alarm communication systems.

## 5 Conclusion

This study demonstrates that the oriental reed warbler exhibits distinct behavioural responses to alarm calls containing different syllable types. Compared to other alarm calls, F alarm calls elicited relatively stronger behavioural responses, attracted a greater number of conspecifics, and induced shorter response latencies. In addition, the frequency and duration of alarm calls can significantly affect the responses of conspecifics. These findings suggest that the oriental reed warbler appears to signal higher levels of urgency by increasing syllable duration and frequency. However, further research is needed to disentangle the independent effects of these acoustic parameters—such as duration, peak frequency, and potentially other features—on receiver behaviour. Moreover, whether the sequence of different syllable types produced in response to predators encodes specific referential or contextual information remains an open question and warrants future investigation.

## Author Contributions

**Qindong Zhou:** data curation (lead), formal analysis (equal), investigation (lead), writing – original draft (equal). **Laikun Ma:** funding acquisition (equal), writing – review and editing (equal). **Wei Guo:** formal analysis (equal). **Jiaojiao Wang:** conceptualization (lead), funding acquisition (equal), methodology (lead), writing – original draft (equal), writing – review and editing (equal). **Longwu Wang:** funding acquisition (equal), writing – review and editing (equal).

## Ethics Statement

The experiments reported here comply with the current laws of China. Fieldwork was carried out under permission from Yongnianwa National Wetland Park. Experimental procedures were in agreement with the Animal Experiment Ethics Committee of Guizhou Normal University (No. 2022001).

## Conflicts of Interest

The authors declare no conflicts of interest.

## Supporting information


**Data S1:** ece372338‐sup‐0001‐DataS1.xlsx.

## Data Availability

The data related to this study have been submitted simultaneously as Supporting Information and are also available from the corresponding author upon reasonable request.
